# Sex, but Not Race, Influences OSA Diagnosis When Applying the 4% Versus 3% Hypopnea Scoring Rule

**DOI:** 10.3390/jcm14248878

**Published:** 2025-12-15

**Authors:** Sandya Subramanian, Annalise E. Miner, Sanford Auerbach, Andrew Spector

**Affiliations:** 1 Boston Medical Center, Department of Neurology, Boston, MA 02118, USA; sandyas@bu.edu; 2 CTE Center, Department of Neurology, Boston University Alzheimer’s Disease Center, Boston, MA 02118, USA; aeminer@bu.edu; 3 Department of Neurology, Duke University School of Medicine, Durham, NC 27710, USA; spector.andrew@gmail.com

**Keywords:** obstructive sleep apnea, hypopnea, sex, race

## Abstract

**Background/Objectives**: Obstructive sleep apnea (OSA) is diagnosed using pulse oximetry, which is less accurate in patients with darker skin. Two hypopnea definitions are in use: the American Academy of Sleep Medicine allows either (A) a 30% airflow decrease with a 3% oxygen desaturation or EEG arousal (“3% Rule”) or (B) a 30% airflow decrease with a 4% oxygen desaturation (“4% Rule”). The Centers for Medicare and Medicaid Services and many payers use only the 4% Rule. We hypothesized that patients from racial minority groups with darker skin tones would be less likely to qualify for OSA treatment using the 4% Rule compared to the 3% Rule. A secondary aim was to examine sex disparities. **Methods**: We reviewed records of adults undergoing overnight sleep studies at an urban academic hospital. Demographics, medical history, and comorbidities were collected. Analyses controlled for age, sex, BMI, anxiety, depression, hypertension, COPD, and smoking. **Results**: A total of 1354 records were analyzed. We found no racial or sex disparities in the proportion of participants who met the 3% but not the 4% Rule. After controlling for covariates, no racial group differed from White participants in meeting only the 3% Rule. However, female participants were significantly less likely than males to meet the 4% Rule. **Conclusions**: Despite known limitations of pulse oximetry associated with skin tone, no racial differences in the diagnosis of OSA were seen in this cohort. However, female patients had lower odds of meeting the 4% Rule, suggesting a potential barrier to treatment.

## 1. Introduction

Obstructive sleep apnea (OSA) is an increasingly common sleep disorder in the United States [1]. The diagnosis of OSA is established by the apnea–hypopnea index (AHI), the sum of an individual’s apneas and hypopneas per hour of sleep. The criteria for defining OSA have evolved over time. Initially, Medicare used a “30 Apnea Rule,” which required 5 apneas per hour over a 6 h period, likely based on initial OSA studies in the 1970s [2]. In 2001, the Clinical Practice Review Committee of the American Academy of Sleep Medicine (AASM) recommended including hypopneas in the measurement of OSA. Hypopneas were initially defined as a 30% reduction in airflow for at least 10 s with a corresponding oxygen desaturation of at least 4% [2]. This definition showed a significant association between AHI and cardiovascular disease, although the committee acknowledged that not all patients who would benefit from therapy would be included in this definition [3].

Today, two definitions of hypopnea are in common use. The AASM Scoring Manual for the Scoring of Sleep and Associated Events: Rules, Terminology, and Technical Specifications defines a hypopnea as (A) a 30% decrease in airflow with either a 3% oxygen desaturation or an EEG arousal (“3% Rule”), or (B) a 30% decrease in airflow with a 4% oxygen desaturation (“4% Rule”) [4]. The Centers for Medicare and Medicaid (CMS) and many commercial payers use only the latter definition of hypopneas [5]. The 3% Rule has more significant associations with certain measures of heart disease, though, such as coronary artery calcification [6], hypertension [7], and diabetes [7]. The 4% Rule is stricter, thus reducing access to sleep apnea treatment. A prior study has shown that using the 4% Rule leads to fewer female patients being diagnosed with OSA compared to the 3% Rule [8]. This creates the potential for disparities in long-term health outcomes among female patients who are unable to access sleep apnea therapy services.

Likewise, reliance on oximetry and the degree of desaturation to diagnose OSA could lead to disparities for patients with different skin tones. Pulse oximeters have the potential to overestimate oxygen saturation and miss episodes of hypoxia at a greater rate in people with darker skin, particularly Black, Hispanic, and Asian individuals, when compared to White individuals [9,10,11]. Given the decreased accuracy of pulse oximeter devices in capturing oxygen saturations of darker-skinned individuals, we hypothesize that people from racial minority backgrounds associated with darker skin tones would be less likely to be eligible for OSA treatment using the 4% Rule than the 3% Rule. Our secondary aim was to confirm the previous finding of a sex disparity due to the use of the 4% Rule. The primary focus of our study is to evaluate how different diagnostic criteria impact which patients qualify for treatment.

## 2. Materials and Methods

**Study Overview:** This study was a retrospective chart review of first-time adult diagnostic in-lab polysomnography studies completed in a single night between January 2022 and May 2023 at Boston Medical Center, an urban academic hospital. Sleep studies could be ordered by any referring provider. Referral indications were not collected, and studies were included regardless of suspected diagnosis. The Boston Medical Center Institutional Review Board approved the study prior to study initiation (IRB Number H-43754).

**Demographics and Medical Information:** Patient records were obtained through the Boston University Clinical Data Warehouse, which identified patients who met these criteria. Patient characteristics (including self-reported race, ethnicity, primary language, sex, and age) and comorbidities (including BMI, smoking status, and presence of hypertension, COPD, depression, and anxiety) were collected through the medical record. Charts were excluded if the sleep study was incomplete or if key variables (such as BMI or comorbidities) could not be obtained. Repeat studies were also excluded.

**Apnea–Hypopnea Index:** All sleep studies were performed in a clinical sleep laboratory accredited by the AASM and were scored using the definitions presented in *The AASM Scoring Manual for the Scoring of Sleep and Associated Events: Rules, Terminology, and Technical Specifications.* Apnea was defined as a drop in the peak signal excursion of at least 90% for at least 10 s [4]. Hypopnea using the 3% Rule was defined as a 30% decrease in airflow with either a 3% oxygen desaturation or an arousal, and hypopnea using the 4% Rule was defined as a 30% decrease in airflow with a 4% oxygen desaturation [4]. Polysomnographic reports were divided into three diagnostic categories: negative study with AHI < 5/h using the 3% rule (“No Sleep Apnea Group”), obstructive sleep apnea diagnosed with an AHI > 5/h by the 3% Rule only and not meeting qualification with the 4% rule (“3% Rule Only Group”), and obstructive sleep apnea diagnosed with an AHI > 5/h using the 4% Rule (“4% Rule Group”). Records were scored by registered polysomnographic technologists and then reviewed by board-certified sleep specialists. Oxygen saturation was measured using Nonin polychromatic pulse oximeters in all patients.

**Statistical Analysis:** Descriptive statistics were used to examine demographics in the entire sample. Models controlled for age, sex, and BMI. Significance was set at a *p*-value of 0.05. Analyses were completed in SPSS (version 29.0.2.0) by IBM (Chicago, IL, USA). Associations of the three diagnostic categories and self-reported racial identity with covariates were analyzed using chi-square tests of independence for binary variables (sex, hypertension, COPD, smoking status, depression, anxiety) and Kruskal–Wallis test for continuous variables (age, BMI, AHI) in separate analyses.

### 2.1. Primary Aim: Differences Between Racial Groups

To answer the question of whether there is a difference in diagnostic categories based on racial group, chi-squared tests of independence were first used to evaluate associations between race and meeting the 3% Rule but not the 4% Rule. Multinomial logistic regression models were then used to examine differences in diagnostic categories across self-identified racial groups. The reference group for the diagnostic category was “no sleep apnea diagnosis” with “White” as the racial reference group. Unadjusted models included only race as the predictor. Adjusted models included race, sex, and covariates (BMI, age, hypertension, COPD, smoking history, depression, and anxiety).

### 2.2. Secondary Aim: Differences Between Sex Groups

To answer the question of whether there is a difference in diagnostic categories based on sex group, chi-squared tests of independence were first performed to evaluate associations between sex and meeting the 3% Rule but not the 4% Rule. Multinomial logistic regression models examined differences in diagnostic categories across sex groups. The reference group for the diagnostic category was “no sleep apnea diagnosis,” with “male” as the sex reference group. Unadjusted models included only sex as the predictor. Adjusted models included sex and covariates (BMI, age, hypertension, COPD, smoking history, depression, and anxiety). The assumption of linearity in the logit for continuous variables (age, BMI) was tested using the Box–Tidwell test. Model fit was assessed using pseudo R^2^ values.

## 3. Results

A total of 1354 medical records were included in the analysis: 455 White patient records, 774 Black patient records, 73 Hispanic patient records, and 52 Asian patient records. 594 patient records were excluded due to missing BMI or comorbidity data.

Participant characteristics, sleep apnea diagnoses, and medical and psychiatric comorbidities by racial group are summarized in Table 1. There were no significant differences in smoking history status between racial groups. White participants had the highest average age, the highest percentage of COPD, and the highest percentage of reported depression and anxiety compared to the other three cohorts. Black participants had the highest average AHI when using both the 3% Rule and the 4% Rule, the highest percentage of women in their cohort, the highest mean BMI, and the highest percentage of hypertension compared to the other three cohorts. Asian participants had the lowest percentage of women in their cohort, the lowest mean BMI, and the lowest percentage of patients with COPD.

### 3.1. Primary Aim: Differences Between Racial Groups

Chi-squared tests showed that the difference in participants who met the 3% Rule but not the 4% Rule was not significantly different between racial groups (*p* = 0.33, Table 2). Unadjusted multinomial logistic regression with race as the predictor showed no difference in meeting only the 3% Rule when Black, Hispanic, and Asian participants were compared to White participants but did show that Black participants were significantly more likely to meet the 4% Rule when compared to no diagnosis (OR 1.369, *p* = 0.013, Table 3).

After controlling for sex, age, BMI, hypertension, depression, anxiety, COPD, and smoking status (Table 3), there continued to be no difference in meeting only the 3% Rule when each racial group was compared to the White group. Additionally, there was no longer a significant difference between the Black and White groups in meeting the 4% Rule.

The assumption of linearity for continuous variables (age, BMI) was tested using the Box–Tidwell test. The assumption of linearity was met for age, both when comparing the 3% Rule Only group to the No Diagnosis group (*p* = 0.326) and when comparing the 4% Rule group to the No Diagnosis group (*p* = 0.519). The assumption of linearity for BMI was met when comparing the 3% Rule Only group to the No Diagnosis group (*p* = 0.136) but showed some nonlinearity when comparing the 4% Rule group to the No Diagnosis group (*p* = 0.032). However, given the large sample size, BMI was retained as a continuous variable in our regression. Pseudo R^2^ values were 0.110 (Cox and Snell) and 0.129 (Nagelkerke), indicating good model fit.

In the multinomial regression, increased age and BMI were significantly associated with increased odds of meeting the 4% Rule (age OR 1.033, *p* < 0.001, Table 3; BMI OR 1.065, *p* < 0.001, Table 3). Additionally, the presence of anxiety was significantly associated with decreased odds of meeting the 4% Rule (OR 0.735, *p* = 0.029, Table 3).

### 3.2. Secondary Aim: Differences Between Sex Groups

Chi-squared tests did not find a significant difference in the proportion of individuals who met the 3% Rule but not the 4% Rule across sex groups (*p* = 0.248, Table 4). Unadjusted multinomial logistic regressions showed no significant difference between male and female participants in meeting only the 3% Rule but did show that female participants were significantly less likely than male participants to meet the 4% Rule (OR 0.588, *p* < 0.001, Table 3). After controlling for age, BMI, hypertension, depression, anxiety, COPD, and smoking, there continued to be no difference between male and female participants in meeting only the 3% Rule, but again, female participants were significantly less likely to meet the 4% Rule (OR 0.482, *p* < 0.001, Table 3).

## 4. Discussion

The different definitions of hypopnea have been shown to have significant effects on the number of people who receive a diagnosis of OSA and thus qualify for insurance coverage for treatment [12,13]. This difference was seen in our study as well, with a significant decrease in the number of patients who qualified using the 4% Rule rather than the 3% Rule within all groups.

In our single-center study, we did not find a significant racial difference in the proportion of participants who were diagnosed with OSA using the 3% Rule but not the 4% Rule. Black patients had higher odds of meeting the 4% Rule than White participants, though this difference did not persist after controlling for covariates and comorbidities. Our findings do not support the conclusion that the use of the 4% Rule instead of the 3% Rule disadvantages Black, Hispanic, or Asian people from receiving a diagnosis of OSA in our patient population. This is surprising given that previous studies have demonstrated that pulse oximetry is less effective in detecting desaturations on darker skin [9,10,11], which would be expected to make it more difficult for races with darker skin, particularly Black but also potentially Hispanic and Asian people, to reach the 4% threshold.

We found that increased age and BMI were associated with increased odds of meeting the 4% Rule, and that the presence of anxiety decreased the odds of meeting the 4% Rule; given that the Black group in this study had the highest BMI and the lowest rate of anxiety, these differences likely account for the unadjusted findings. While there were significantly different rates of other comorbidities that have been shown to be associated with OSA, such as COPD [14] and hypertension [15], between racial groups in this study, these factors were not seen to affect the odds of meeting only the 3% Rule or the 4% Rule between racial groups.

While we did not find an effect of the 3% versus 4% rule on Black versus White patients in this patient group, it is possible that the impact of the oximeter bias was not detected due to the increased underlying severity of OSA in Black patients, such that they overcame the technological bias and qualified for a 4% Rule diagnosis anyway. Black men, for example, have been shown to have increased severity of OSA [16], and Black individuals present with increased symptom burden [17]. In our study, Black patients had the highest average AHI of all racial groups when using both the 3% Rule and the 4% Rule, indicating that this group in our sample had greater OSA severity. Additionally, given that our patient sample was drawn from patients undergoing inpatient polysomnography at a tertiary care center, it is possible that the racial minority patient groups in our study may represent patients with more advanced OSA, limiting our ability to include milder cases, as such cases may not be referred for a sleep study.

In addition to not finding a racial disparity in meeting only the 3% Rule, we also did not find a sex difference. However, we did find the female group had significantly lower odds of meeting the 4% Rule when compared to the male group, aligning with prior work [8]. There are physiological differences between the sexes that could contribute to this observed disparity. The upper airways of women tend to be less collapsible, and women tend to have lower loop gain, leading to fewer apneas and thus lower AHIs [18]. Additionally, prior work has shown that women tend to have higher AHIs in REM sleep [19], but this can be masked in overall AHI, as we could not calculate REM-specific AHI in our study.

A strength of our study is that all the included data came from adult patients who underwent their first, one-time, overnight inpatient sleep study, allowing for a controlled environment between all patients. Scoring criteria were standardized between patients, allowing for more accurate AHI reporting, and we were able to obtain and control for important covariates and comorbidities. Additionally, the pulse oximeters used in this study were polychromatic, which have been shown to overestimate oxygen saturation in individuals with darker skin to a greater degree than monochromatic oximeters [20]; thus, it is particularly meaningful that we did not find a racial difference in our sample population. However, a major limitation was that our sample came from one urban academic hospital, which may not be reflective of other patient groups. Data was obtained retrospectively, which did not allow us to control for all sleep study variables, such as sleep position, medication use, or environmental factors such as noise, which could lead to confounding.

Larger, multi-center, prospective studies are needed to validate these findings to ensure that health policy regarding OSA is equitable. In addition, further work must be performed to improve pulse oximetry to ensure the tools used to diagnose OSA are effective in all patient populations [20]. While the use of the 4% Rule compared to the 3% leads to a significant number of patients being excluded from OSA treatment, reassuringly, this does not appear to discriminate by race in our cohort. Female patients, on the other hand, are likely disadvantaged by the 4% rule. To increase access to treatment and ensure equitable healthcare, the 4% Rule should be retired in favor of the 3% Rule.

## Figures and Tables

**Table 1:** Participant Characteristics.

	Overall (*n* = 1354)	White (*n* = 455)	Black (*n* = 774)	Hispanic (*n* = 73)	Asian (*n* = 52)	*p*-Value
Demographics						
Age (years) ^1^	51.37 (14.80)	51.77 (14.68)	51.74 (14.56)	45.55 (13.90)	50.54 (18.72)	0.004 ^2^
Sex						
Female n (%)	729 (54)	225 (49)	449 (58)	35 (48)	20 (38)	0.002 ^3^
BMI ^1^	35.36 (8.46)	33.91 (7.58)	36.52 (8.83)	35.61 (8.34)	30.49 (6.58)	<0.001 ^2^
AHI						
Using 3% Rule ^1^	22.8 (26.17)	19.46 (23.07)	24.71 (28.1)	23.33 (24.27)	21.82 (20.58)	0.047 ^2^
Using 4% Rule ^1^	17.6 (24.49)	14.22 (21.01)	19.57 (26.5)	18.15 (22.96)	16.74 (19.77)	0.009 ^2^
Medical Comorbidities						
Hypertension *n* (%)	778 (57)	208 (46)	508 (66)	31 (42)	31 (60)	<0.001 ^3^
COPD *n* (%)	89 (6.6)	43 (9.5)	42 (5.4)	3 (4.1)	1 (1.9)	0.016 ^3^
Smoking *n* (%)	44 (3.2)	21 (4.6)	20 (2.6)	2 (2.7)	1 (1.9)	0.247 ^3^
Psychiatric Comorbidities						
Depression *n* (%)	511 (38)	193 (42)	294 (38)	14 (19)	10 (19)	<0.001 ^3^
Anxiety *n* (%)	412 (30)	197 (43)	181 (23)	22 (30)	12 (23)	<0.001 ^3^

^1^ Mean (SD). ^2^ Kruskal–Wallis rank sum test. ^3^ Pearson’s Chi-squared test. AHI = Apnea–hypopnea index. BMI = Body mass index. COPD = Chronic obstructive pulmonary disease. Dx = Diagnosis.

**Table 2:** Frequency of AHI Category by Race.

Racial Identity Group	No Diagnosis (%)	3% Rule but Not 4% Rule (%)	4% Rule (%)
White	45.5	11.9	42.6
Black	39.3	10.3	50.4
Hispanic	42.5	9.6	47.9
Asian	42.3	3.9	53.8
		χ^2^ = 3.42, *p*-value = 0.33	

χ^2^—Chi-squared test statistic. % = Percentage.

**Table 3:** Multinomial Regression Models.

A. Self-Reported Racial Identity Multinomial Regression Models				
**Predictor**	**Unadjusted 3% Rule Only OR (*p*)**	**Unadjusted 4% Rule OR (*p*)**	**Adjusted 3% Rule Only OR (*p*)**	**Adjusted 4% Rule OR (*p*)**
Black Participants	1.009 (0.965)	**1.369 (0.013)**	1.097 (0.658)	1.205 (0.181)
Hispanic Participants	0.866 (0.746)	1.205 (0.484)	0.934 (0.881)	1.275 (0.396)
Asian Participants	0.348 (0.162)	1.358 (0.311)	0.396 (0.223)	1.580 (0.157)
Female Sex	--	--	0.812 (0.303)	**0.482 (<0.001)**
Age	--	--	1.008 (0.312)	**1.033 (<0.001)**
BMI	--	**--**	1.012 (0.348)	**1.065 (<0.001)**
Hypertension	--	--	0.771 (0.234)	0.911 (0.509)
Depression	--	--	1.181 (0.404)	1.065 (0.636)
Anxiety	--	--	1.245 (0.285)	**0.735 (0.029)**
COPD	--	--	0.959 (0.909)	0.607 (0.05)
Smoking	--	--	0.553 (0.348)	0.998 (0.994)
**B. Sex Group Multinomial Regression Models**				
**Predictor**	**Unadjusted 3% Rule Only OR (*p*)**	**Unadjusted 4% Rule OR (*p*)**	**Adjusted 3% Rule Only OR (*p*)**	**Adjusted 4% Rule OR (*p*)**
Female Sex	0.938 (0.737)	**0.588 (<0.001)**	0.823 (0.333)	**0.489 (<0.001)**
Age	--	--	1.008 (0.275)	**1.032 (<0.001)**
BMI	--	**--**	1.014 (0.265)	**1.065 (<0.001)**
Hypertension	--	--	0.782 (0.250)	0.944 (0.678)
Depression	--	--	1.204 (0.351)	1.049 (0.719)
Anxiety	--	--	1.228 (0.307)	**0.708 (0.013)**
COPD	--	--	0.96 (0.913)	**0.592 (0.039)**
Smoking	--	--	0.557 (0.352)	0.975 (0.939)

Unadjusted and adjusted multinomial regression models examined (**A**) the association of self-identified racial group with the 3% Rule Only and 4% Rule and (**B)** the association of sex group with the 3% Rule Only and the 4% Rule. The reference groups used for this logistic regression include ‘No Diagnosis’ for AHI category, ‘White’ for self-reported racial group, and ‘Male’ for sex. Odds ratios represent the likelihood of meeting each diagnostic criterion relative to no criteria met. Adjusted models control for presence of additional covariates (age, BMI, hypertension, depression, anxiety, COPD, and smoking status). Bolded values indicate significance of *p* ≤ 0.05. AHI = Apnea–Hypopnea Index. Dx = Diagnosis. BMI = Body mass index. COPD = Chronic obstructive pulmonary disease.

**Table 4:** Frequency of AHI Category by Sex.

Sex Category	No Diagnosis (%)	3% Rule but Not 4% Rule (%)	4% Rule (%)
Male	35.8	9.4	54.7
Female	46.6	11.5	41.8
		χ^2^ = 1.33, *p*-value = 0.248	

χ^2^—Chi-squared test statistic. % = Percentage.

## Data Availability

The data that support the findings of this study are available from the author, Sandya Subramanian, upon reasonable request.

## Abbreviations

The following abbreviations are used in this manuscript:

AASM	American Academy of Sleep Medicine
AHI	Apnea–hypopnea index
BMI	Body mass index
CMS	Centers for Medicare and Medicaid
COPD	Chronic obstructive pulmonary disease
OSA	Obstructive sleep apnea
